# Cilengitide induces cellular detachment and apoptosis in endothelial and glioma cells mediated by inhibition of FAK/src/AKT pathway

**DOI:** 10.1186/1756-9966-27-86

**Published:** 2008-12-29

**Authors:** Leticia Oliveira-Ferrer, Jessica Hauschild, Walter Fiedler, Carsten Bokemeyer, Johannes Nippgen, Ilhan Celik, Gunter Schuch

**Affiliations:** 1 Department of Oncology and Hematology with Section Pneumology, University Medical Center Hamburg-Eppendorf, Hamburg, Germany; 2 Merck Serono, Darmstadt, Germany

## Abstract

**Background:**

The antiangiogenic agent cilengitide disrupts integrin binding to the extracellular matrix leading to apoptosis of activated endothelial cells. Integrins are also widely expressed in malignant glioma and integrin inhibitors may directly target tumor cells in this disease. Aim of the current study was to investigate effects of cilengitide on endothelial and glioma cells on molecular and cellular levels.

**Results:**

Cilengitide caused dose-dependent detachment of endothelial cells from cell culture dishes. Proliferation of endothelial cells was significantly inhibited while the proportion of apoptotic cells was increased. Incubation of integrin-expressing glioma cells with cilengitide caused rounding and detachment after 24 hours as observed with endothelial cells. Cilengitide inhibited proliferation and induced apoptosis in glioma cells with methylated MGMT promotor when given alone or in combination with temozolomide. In endothelial as well as glioma cells cilengitide inhibited phosphorylation of FAK, Src and Akt. Assembly of cytoskeleton and tight junctions was heavily disturbed in both cell types.

**Conclusion:**

Cilengitide inhibits integrin-dependent signaling, causes disassembly of cytoskeleton, cellular detachment and induction of apoptosis in endothelial and glioma cells thereby explaining the profound activity of integrin inhibitors in gliomas. The combination of cilengitide with temozolomide exerted additive effects in glioma cells as observed clinically.

## Background

Angiogenesis, the formation of blood vessels from pre-existing vasculature, has been identified as an essential mechanism in tumor growth [1]. This process is mediated by proangiogenic growth factors such as vascular endothelial growth factor (VEGF) inducing proliferation, migration and tube formation of endothelial cells [2]. Another important feature is the interaction of endothelial cells with surrounding extracellular matrix (ECM) that is mediated by integrins. Integrins are transmembrane receptors composed of two subunits binding to ECM and base membrane proteins [3]. Integrin binding mediates adhesion to surrounding structures and regulates cell survival, growth and mobility [4]. Of more than 20 known α/β heterodimers the integrins αvβ3 and αvβ5 are predominantly expressed in proangiogenic endothelial cells [5,6]. A variety of blocking agents and antibodies targeting either one or both integrins has been developed for antiangiogenic therapy. Cilengitide, a cyclic pentapeptide mimicking the Arg-Gly-Asp (RGD) binding site, was identified as a potent and selective integrin antagonist [7] inhibiting binding to ECM components of αvβ3 and αvβ5 integrins. It was shown to inhibit VEGF and bFGF-induced migration and tube formation in vitro [8]. Cilengitide inhibits proliferation and differentiation of endothelial progenitor cells playing an important role in neoangiogenesis in cancer [9]. In preclinical models, cilengitide was synergistic with radioimmunotherapy in breast cancer and orthotopic brain tumor models [10,11].

Expression of αvβ3 and αvβ5 integrins is not restricted to activated endothelial cells. Especially brain tumors are known to widely express these integrin family members in tumor cells [12-14]. Labelled integrin antibodies have been used for tumor imaging in glioma models *in vivo *[15] and cilengitide as well as other inhibitors have been successfully tested in preclinical models of glioma [16,17]. While failing in a large trial of pancreatic cancer [18], cilengitide has been shown to be active in malignant glioma given alone [19,20] or in combination with chemotherapy [21]. However, additive activity of the combination of cilengitide with temozolomide was seen only in patients with methylated promotor of O6-methylguanine DNA methyltransferase (MGMT), so far known as a predicitve marker for temozolomide therapy.

Direct effects of integrin inhibition on brain tumors were suggested from antisense experiments in medulloblastoma cell lines where growth inhibition and induction of apoptosis was observed [22]. In vitro, cilengitide caused detachment of U87 and DAOY cells with consecutive apoptosis induction depending on the matrix used [23]. However, no further data on signaling effects of cilengitide either cell type have been shown so far. Therefore, the current study was performed to investigate the morphological and molecular mechanisms induced by cilengitide in endothelial and in glioma cells.

## Methods

### Cell culture and Reagents

Human microvascular endothelial cells (HMEC-1), kind gift from Centre for Disease Control and Prevention, Atlanta, U.S.A., were grown in MCDB 131-medium (Gibco) supplemented with 5% fetal bovine serum (FBS, Gibco), 2 mM L-glutamine (Gibco), 10 ng/ml epidermal growth factor (ICN, Costa Mesa, CA, U.S.A.) and 1 μg/ml hydrocortisone (ICN), and maintained on uncoated dishes in a 5% CO_2_/95% air atmosphere in a humidified incubator at 37°C. Porcine aortic endothelial cells stably transfected with KDR (PAE-KDR), provided by Shay Soker, Winston-Salem, NC, were maintained in F-12/HAM medium supplemented with 5% fetal bovine serum at 37°C in 5% CO_2_/95% air. Commercial human umbilical vein endothelial cells (HUVECs) (Lonza) were cultured in EGM-2 medium (Clonetics) including 2% fetal calf serum. The human glioblastoma cell lines G28 and G44 [24,25], kindly provided from the Department of Neurosurgery, University Hospital Hamburg-Eppendorf, were cultured in Modified Eagle's Medium supplemented with 10% fetal bovine serum on uncoated dishes. Cilengitide (CGT) was kindly provided by Merck Serono, Darmstadt, Germany. Stock solutions were diluted in sterile physiological saline solution at 20 mg/ml. Cells were incubated with cilengitide in final concentrations of 1, 5 and 50 μg/ml. Temozolomide (TMZ) was purchased from Bristol Myers Squibb, Munich. Stock solution was diluted in DMSO at 5 mg/ml. Cells were treated with temozolomide in a final concentration of 5 μg/ml. Texas Red-X phalloidin was from Invitrogen, mouse monoclonal anti phospho-Akt (Ser473) antibody was from Cell Signaling, rabbit polyclonal anti phospho-Src (Y418) antibody was from Biosource, mouse monoclonal anti phospho-Src (Y416) was from Biomol, mouse monoclonal anti Src antibody was from Upstate (NY, USA), mouse monoclonal anti phospho-FAK (Y397) was from BD Biosciences, rabbit polyclonal anti ZO-1, anti Erk1/2, mouse monoclonal anti phospho-Erk and anti β-actin antibodies were from Santa Cruz Biotechnology.

Tissue culture plates were incubated with a 12 mg/ml polyHEMA (Poly(2-hydroxyethyl methacrylate); Sigma Aldrich) ethanol solution at 37°C or with 10 μg/ml fibronectin (Harbor Bio-Products, Norwood, MA, USA) at 4°C overnight when indicated.

### Proliferation

HMEC-1, G28 and G44 cells (1 × 10^4 ^per well) were seeded on uncoated 48 well plates and incubated in serum free medium, medium containing 4% FCS or medium containing 4% FCS with cilengitide (1, 5 and 50 μg/ml) or/and temozolomide (5 μg/ml). For experiments with temozolomide, control cells were treated with medium containing 4% FCS and DMSO at the equivalent concentration used for the temozolomide stock solution. Each stimulation was performed in triplicate. After incubation for 24, 48 and 72 hours at 37°C cells were trypsinized and counted.

### Apoptosis

HMEC-1, G28 and G44 cells (5 × 10^5^) were incubated on uncoated dishes with and without cilengitide (1, 5 and 50 μg/ml) for 24 hours at 37°C. G28 and G44 cells (2 × 10^5^) were incubated with temozolomide (5 μg/ml) and cilengitide (5 μg/ml) in combination or separately for 48 hours at 37°C. For experiments with temozolomide, control cells were treated with medium containing 4% FCS and DMSO at the equivalent concentration used for the temozolomide stock solution. Apoptosis was assessed after staining with FITC-labeled annexin-V and PI (BD Pharmingen) by flow cytometric analysis. Positive staining with FITC-labeled annexin-V reflects a shift of phosphatidylserine from the inner to the outer layer of the cytoplasmatic membrane, which occurs early in apoptosis. Annexin-V-positive and PI-negative cells were scored as early apoptotic cells. Cells labeled by annexin V and PI have been determined as late apoptotic. Annexin negative and PI-positive events display necrotic cells.

### Immunofluorescence Analysis

HMEC-1 and G28 cells were plated on cover slips and treated with and without cilengitide (1, 5 and 50 μg/ml) for 1 hour at 37°C. Cells were fixed with 4% paraformaldehyde, permeabilized with methanol, and stained for ZO-1 (Santa Cruz Biotechnology) and actin (PE-phalloidin, Invitrogen). Immunofluorescence analysis was carried out with an Axioplan (Zeiss) inverted microscope.

### Western blotting

Protein extracts were prepared with lysis buffer solution containing 50 mM Tris-HCl pH 7,4, 150 mM NaCl, 100 mM EGTA, 1% Nonidet P-40, 10% Na-deoxycholate, 1× protease inhibitor cocktail (Sigma Aldrich) and 1 mM sodium orthovanadate. Protein lysates were boiled in SDS-sample buffer before being applied into a 10% SDS-PAGE. After electrotransfer to nitrocellulose membranes (Schleicher & Schuell, Dassel, Germany) and blocking in TBS-T buffer containing 5% non-fat milk overnight, blots were incubated with the appropriate primary antibody. The subsequent incubation with the peroxidase-conjugated secondary antibodies was followed by detection using ECL Western blotting detection reagents (Amersham). Specific bands were quantified by densitometric analysis using the GS-800 Calibrated Densitometer and Quantity-one software (BioRad).

### RNA isolation, reverse transcription and RT-PCR

Total cellular RNA from HMEC-1, G28 and G44 cells was extracted using RNeasy Kit from Qiagen. 3 μg of total RNA each were reverse transcribed into cDNA using the You-Prime First-Strand cDNA synthesis kit (Amersham). Following primers were used to amplify the genes encoding integrin subunits αv and β3: integrin αv (671 bp) forward (5'-cttcaacctagacgtggacagt-3') and reverse (5'-ttgaaatctccgacagccacag-3'), integrin β3 (123 bp) forward (5'-agaagagccagagtgtccca-3') and reverse (5'-gaattcttttcggtcgagga-3'). For amplification, touch down-PCR was performed (one cycle from 65°C-55°C and 25 cycles at 55°C). The amplification products were visualized on a 1% ethidium bromide-stained agarose gel.

### Methylation-specific PCR

DNA was extracted using QIAmp DNA Mini Kit from Qiagen. 2 μg genomic DNA was denaturated and chemical modificated via bisulfite treatment using the EZ DNA Methylation-Gold Kit from Zymo Research. For glioma cell lines (G28 and G44) and normal lymphocytes DNA was first amplified with flanking PCR primers as previously described [26]. The resulting fragment was used as a template for the MSP reaction. Subsequent PCR was performed with specific primers for either methylated or the modified unmethylated promotor region of MGMT gene. Primer sequences for the unmethlyated reaction were: 5'-TTT GTG TTT TGA TGT TTG TAG GTT TTT GT-3' (upper primer) and 5'-AAC TCC ACA CTC TTC CAA AAA CAA AAC A-3' (lower primer) and for the methylated reaction 5'-TTT CGA CGT TCG TAG GTT TTC GC-3' (upper primer) and 5'-GCA CTC TTC CGA AAA CGA AAC G-3' (lower primer). The annealing temperature was 59°C. Universal methylated human DNA Standard was used as a positive control for methylated alleles of MGMT and DNA from normal lymphocytes was used as a negative control. The PCR products were separated on a 4% agarose gel.

## Results

### Effect of cilengitide on endothelial cells

**Figure 1:**
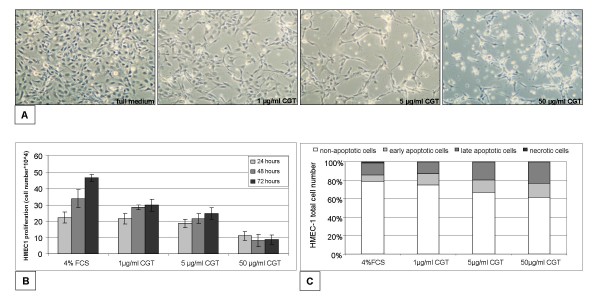
**Effects of cilengitide on endothelial cell proliferation and apoptosis**. (A) Adhesion and morphology. HMEC-1 endothelial cells grown on uncoated dishes were treated for 24 hours with control medium or cilengitide at concentrations of 1 μg/ml, 5 μg/ml and 50 μg/ml. Phase contrast micrographs were taken 24 hours after treatment. (B) Proliferation assay. Cells were treated with medium containing 4% FCS and cilengitide at the concentrations indicated, trypsinized and counted after 24, 48 and 72 hours incubation at 37°C. (C) Apoptosis. Floating and attached cells were collected 24 hours after treatment with cilengitide at the indicated concentrations. Apoptosis was assessed by flow cytometry after annexin V and propidium iodide staining. Experiments were performed in triplicate.

We first studied the effect of cilengitide on endothelial cell attachment *in vitro*. The human microvascular endothelial cell line HMEC-1 cultured in monolayer on uncoated dishes was incubated with and without cilengitide at concentrations of 1, 5 and 50 μg/ml. As shown in figure 1A, cilengitide induced a dose dependent detachment of HMEC-1 cells, accompanied by striking morphologic changes after 24 hours incubation.

### Cilengitide inhibits proliferation and induces apoptosis in endothelial cells

Cilengitide, added at concentrations of 1, 5 and 50 μg/ml over a period of 72 hours, significantly decreased proliferation of HMEC-1 cells grown on uncoated dishes *in vitro*. We observed a dose-dependent reduction of endothelial cell counts, as shown in figure 1B. At a concentration of 1 μg/ml, cilengitide induced 33%, 59% and 44% inhibition after 24, 48 and 72 hours, respectively. In contrast, at concentrations of 5 and 50 μg/ml almost no proliferation of endothelial cells was observed comparable to the effect of serum starvation.

To investigate whether apoptosis was responsible for the decrease of adherent endothelial cells treated with cilengitide, we measured Annexin V/propidium iodide (PI) positive cells after incubation with and without cilengitide at varying concentrations. In HMEC-1 cells cilengitide had a significant pro-apoptotic effect, which was more profound with increasing concentrations (1, 5 and 50 μg/ml) after 24 hours incubation (figure 1C).

### Effect of Cilengitide on glioma cells

**Figure 2:**
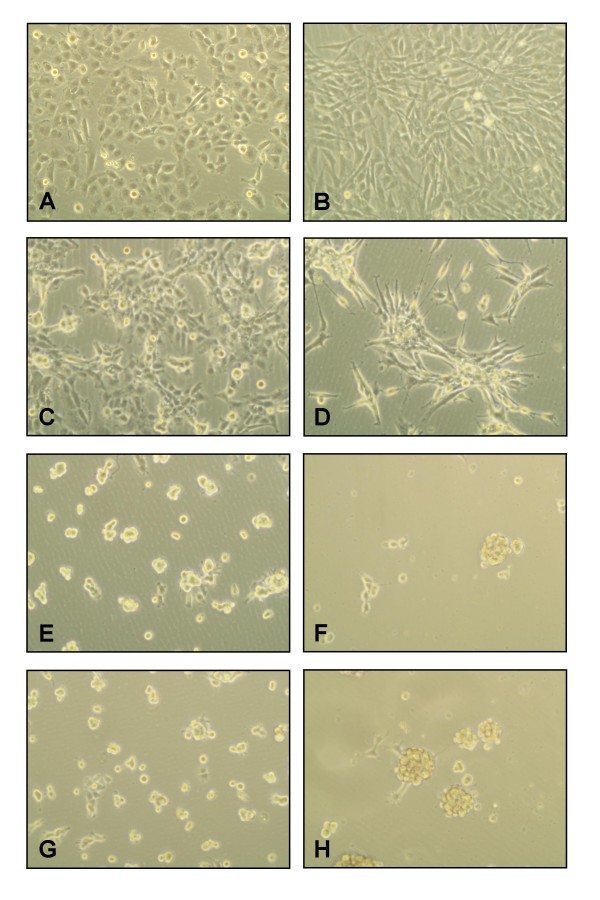
**Effects of cilengitide on cell adhesion and morphology of glioma cell lines G28 and G44**. Cell lines G28 (left panel) and G44 (right panel) were treated with full medium (A, B) or cilengitide at concentrations of 1 μg/ml (C, D), 5 μg/ml (E. F) and 50 μg/ml (G, H) in full medium. Phase contrast micrographs were taken 24 hours after treatment.

Cilengitide has been reported to inhibit glioblastoma growth via suppressing angiogenesis [16]. Since cilengitide acts as antagonist to integrin αvβ3 and αvβ5 and both integrins are expressed in glioma cells, especially on the periphery of high-grade gliomas [13], we asked whether cilengitide has a direct effect on glioma cells. Human glioma cell lines G28 and G44 expressing integrins αvβ3 and αvβ5 (data not shown) were incubated with increasing concentrations of cilengitide and changes were studied after 24 hours. Similar to endothelial cells, cilengitide inhibited adhesion of G44 and G28 glioma cells in a dose-dependent manner (fig. 2). In contrast to control cell cultures, where few cells detached (fig. 2A–2B), G28 (fig. 2C) and G44 (fig. 2D) cells treated with 1 μg/ml cilengitide for 24 hours showed striking cellular detachment. After 24 hours exposure to 5 μg/ml (fig. 2E–2F) and 50 μg/ml (fig. 2G–2H) cilengitide, almost all cells were rounded up, completely detached and seemed to form cell clusters.

### Cilengitide inhibits proliferation and induces apoptosis in glioma cells

**Figure 3:**
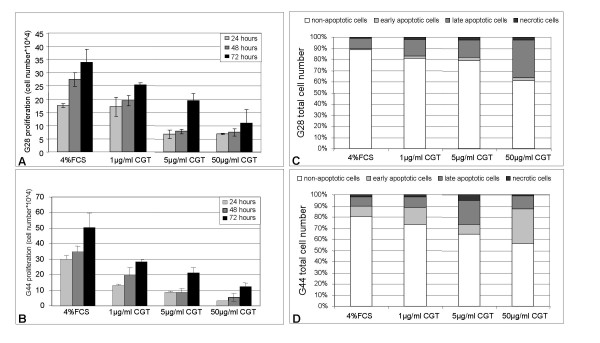
**Cilengitide inhibits proliferation and induces apoptosis in glioma cell lines G28 and G44**. Proliferation assay. Cell lines G28 (A) and G44 (B) were treated with control medium or with medium containing cilengitide at the concentrations indicated and then trypsinized and counted after 24, 48 and 72 hours incubation at 37°C. Apoptosis. Floating and attached G28 (C) and G44 (D) cells were collected 24 hours after treatment with cilengitide at the indicated concentrations. Apoptosis was assessed by flow cytometry after annexin V and propidium iodide staining.

Glioma cell lines G28 and G44 were treated with 1, 5 and 50 μg/ml cilengitide and cell counts were determined after 24, 48 and 72 hours. We observed an inhibitory effect on cell proliferation already at the lowest concentration (1 μg/ml) with significant differences becoming visible after 48 hours (fig. 3A–3B). Higher concentrations of cilengitide abolished cell proliferation and induced apoptosis already after 24 hours exposure to the drug. Annexin V/propidium iodide staining revealed the presence of 18% apoptotic G28 and 30% apoptotic G44 cells after treatment with 5 μg/ml cilengitide. At a concentration of 50 μg/ml, 35% – 50% of G28 and G44 cells respectively underwent apoptosis showing a dose dependent apoptosis induction in glioma cells (fig. 3C–3D).

**Figure 4:**
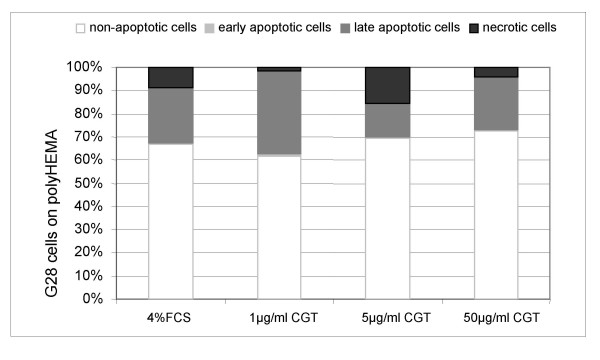
**Cilengitide induces anoikis in G28 glioma cells**. Cilengitide was added to G28 cells at the time of seeding on polyHEMA. Floating and attached cells were collected 24 hours after treatment with cilengitide at the indicated concentrations. Apoptosis was assessed by flow cytometry after annexin V and propidium iodide staining. There was no difference in rates of apoptotic cells between groups. This experiment indicates that the effect of cilengitide is comparable to other conditions inducing anoikis.

In contrast to integrin-mediated cell death of adherent cells, apoptosis of cells loosing adherence is termed anoikis [27]. To compare cilengitide-induced apoptosis with anoikis, G28 cells were seeded on polyHEMA-coated surfaces that totally prevent cell adhesion. The rate of apoptotic cells when G28 were cultured on polyHEMA was identical to those observed with cilengitide treatment of adherent growing cells (fig. 3C). When cilengitide was added to non-adherent cells no increase in the rate of apoptosis was observed (fig. 4). Therefore, the mechanism of cilengitide-mediated apoptosis was indistinguishable from anoikis, suggesting that the anti-adhesive effect of cilengitide is responsible for apoptosis induction.

### Cilengitide inhibits FAK, Src and VEGF-induced ERK1/2 phosphorylation in endothelial cells

**Figure 5:**
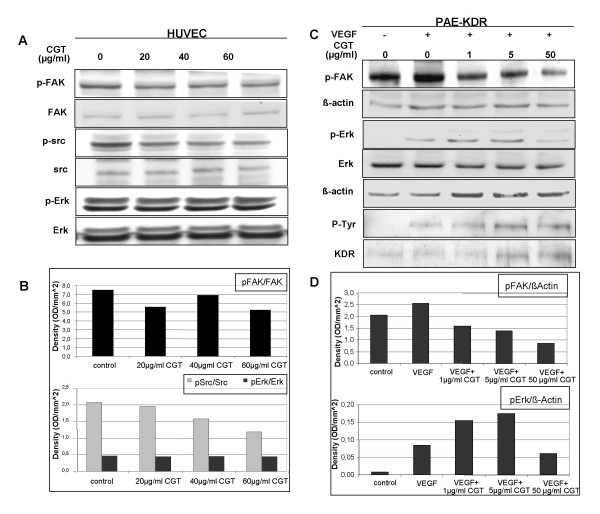
**Cilengitide inhibits FAK and Src but not VEGF-induced phosphorylation of KDR and ERK1/2 in endothelial cells**. (A) Western blotting and (B) densitometric analysis of FAK, Src and Erk1/2 in HUVEC. Cells were maintained 24 hours in serum-free medium on uncoated dishes and then treated with cilengitide for 1 hour at concentrations indicated. Cell lysates were separated by SDS-PAGE and transferred to a nitrocellulose membrane to detect phosphorylated FAK, Src and Erk1/2. (C) Western blotting analysis of FAK, KDR and Erk1/2 and (D) densitometric analysis of FAK and Erk1/2 in PAE-KDR cells. Cells were maintained 24 hours in serum-free medium, treated with VEGF (5 ng/ml) and cilengitide at concentrations indicated and incubated 30 minutes on ice and 7 minutes at 37°C. For detection of FAK and Erk1/2, cell lysates containing same amount of protein were separated by SDS-PAGE and transferred to a nitrocellulose membrane. For detection of KDR, cells were extracted and subjected to KDR immunoprecipitation. Total proteins from immunoprecipitates were separated by SDS-PAGE and transferred to a nitrocellulose membrane. Thereafter, membranes were probed with specific antibodies against phosphorylated FAK, KDR and Erk1/2.

Cilengitide has been shown to inhibit proliferation and to induce apoptosis in endothelial cells, but its effects on integrin mediated signaling pathways are unknown. Therefore we studied the effect of integrin blockade by cilengitide on the phosphorylation and thus activation of p38 MAPK, p44/42 MAPK (Erk1/2), FAK, Src and Akt. After 24 h of starvation in serum-free medium HUVECs grown on uncoated dishes were treated with 20, 40 and 60 μg/ml cilengitide for 1 hour and the activation of signaling molecules was evaluated by Western blotting with phosphoprotein-specific antibodies. As shown in figure 5A, integrin blockade moderately reduced phosphorylation of FAK and Src dose-dependently, but not of Erk 1/2 and p38 (data not shown) under this conditions. Densitometric analyses were performed to quantify the effects on signalling events (figure 5B).

Integrins can physically interact with growth factor receptors to regulate a variety of biological processes. In particular, the interaction between αvβ3 integrin and KDR is important for downstream signaling of both receptor types [28,29]. Given that cilengitide interacts with the extracellular domain of αvβ3, and might thereby interfere with the association of KDR with αvβ3 integrin, we examined whether cilengitide also affects downstream components of KDR signaling pathways. Stimulation of PAE-KDR cells (porcine aortic endothelial cell line stably transfected with VEGFR-2/KDR) with VEGF induced phosphorylation of KDR, FAK and Erk. Simultaneous treatment of PAE-KDR cells with VEGF and cilengitide (1, 5 and 50 μg/ml) did not alter KDR activation when comparing to total KDR protein but inhibited phosphorylation of FAK (figure 5C/D). Erk phosphorylation was decreased only at higher concentrations of cilengitide (50 μg/ml) after 10 minutes, which may be due to pathway crosstalk, since KDR activity was not inhibited under these conditions. These results suggest that cilengitide inhibits integrin-dependent signaling through FAK and Src while it does not influence VEGFR-2/KDR and its downstream pathways in endothelial cells.

### Cilengitide inhibits phosphorylation of FAK, Src and Akt in glioma cells

**Figure 6:**
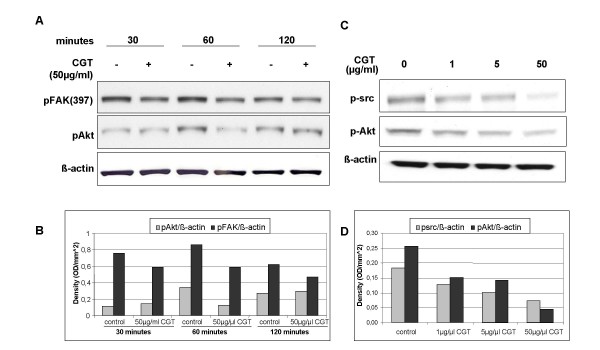
**Cilengitide inhibits phosphorylation of FAK, Src and Akt in glioma cells**. G28 cells were treated with 50 μg/ml cilengitide for 30, 60 and 120 minutes at 37°C. Cell lysates containing similar amounts of protein were separated by SDS-PAGE, transferred to a nitrocellulose membrane and probed with specific antibodies for detection of β-actin and phosphorylated FAK and Akt (A). pFAK and pAkt were quantified by densitometry (OD/mm^2^) (B). G28 cells were treated with cilengitide at the concentrations indicated for 1 hour at 37°C. Cell lysates were separated by SDS-PAGE and transferred to a nitrocellulose membrane to detect β-actin and phosphorylated Src and Akt (C). pSrc and pAkt were then quantified by densitometry (OD/mm^2 ^(D).

We next studied the effect of cilengitide on integrin-mediated signaling pathways in the absence of VEGF in glioma cells. In order to determine the optimal time frame for signaling events G28 cells were lyzed after 30, 60 and 120 minutes of incubation with cilengitide (50 μg/ml) and analyzed for activation of FAK and Akt by Western blot using phospho-specific antibodies. As shown in figure 6A and 6B, inhibition of FAK phosphorylation by cilengitide was observed already at 30 minutes and this effect continued for at least until 1 hour. Additionally, inhibition of pAkt was noted after 60 minutes. Therefore, following experiments were performed with incubations of 1 hour using increasing concentrations of cilengitide. Incubation with cilengitide under these conditions caused inhibition of Src and Akt phosphorylation downstream of FAK in a dose dependent manner as shown in figure 6C. Results were quantified using densitometric analyses (figure 6D).

These results demonstrate that cilengitide inhibit identical pathways in glioma and endothelial cells explaining similar effects such as detachment and apoptosis induction observed in both cell types.

### Cilengitide induces disassembly of tight junctions and actin cytoskeleton

To analyze the effect of cilengitide on the distribution of the tight junction proteins and actin filaments, we performed immunofluorescent staining of endothelial (HMEC-1) and glioma cells (G28) for zona occludens (ZO-1) and phalloidin.

**Figure 7:**
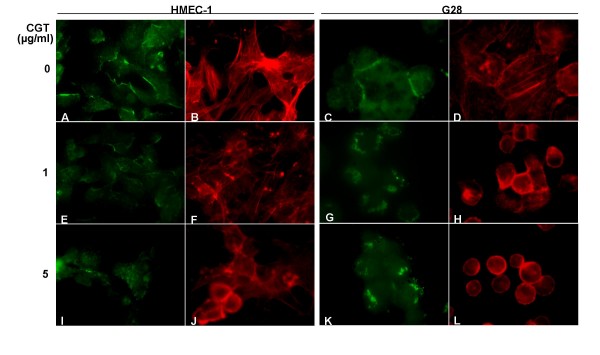
**Cilengitide induces disassembly of tight junctions and actin cytoskeleton in endothelial and glioma cells**. HMEC-1 (left panel) or G28 cells (right panel) were treated with full medium (A-D) or cilengitide at concentrations of 1 μg/ml (E-H) and 5 μg/ml (I-L) in full medium for 24 hours. Cells were then fixed and stained for ZO-1 (green) and actin (red).

In control cells staining with ZO-1 highlighted tight junctions at cellular borders with continuous staining along cell-cell contacts on both HMEC-1 and G28 cells (fig. 7A, 7C). Cells exposed to cilengitide for 24 hours lost distribution of ZO-1 to intercellular junctions, showing irregular distribution with sporadic cytoplasmatic aggregates (figures 7E, 7I, 7G, 7K).

PE-phalloidin staining of actin cytoskeleton, revealed a disassembly of actin filaments in cilengitide treated endothelial and glioma cells compared to controls (figures 7B, 7D). With the disappearance of the actin fibers from the cell interior, we observed clustering of microfilaments along cell borders (figures 7F, 7J, 7H, 7L). Although effects were similar in both cell types, glioma cells appeared more sensitive for disassembly of filaments and cellular detachment. These observations highlight the profound changes on intercellular contacts and cytoskeleton caused by cilengitide similarly in endothelial and glioma cells.

### MGMT promotor methylation status of glioma cell lines

**Figure 8:**
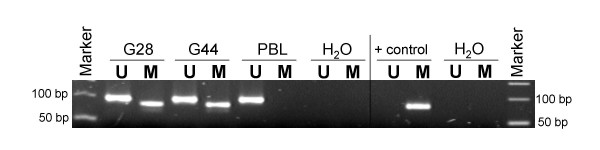
**Methylation status of MGMT promotor in glioma cell lines G28 and G44**. A methylation-specific PCR assay was used with universal methylated human DNA Standard as a positive control for methylation, peripheral blood lymphocytes (PBL) as a negative control for methylation and water as a negative PCR control. U denotes the presence of unmethylated genes and M the presence of methylated genes. Both glioma cell lines G28 and G44 show methylation.

Temozolomide (TMZ) is a DNA-methylating agent with activity as monotherapy in malignant gliomas [30-32]. However the benefit from temozolomide treatment in glioblastoma is strongly associated with MGMT promoter methylation [33]. Therefore, we determined the MGMT promotor methylation status using a methylation-specific PCR assay. Cell lines G28 and 44 both had a methylated MGMT promotor as shown in figure 8. Lymphocytes from peripheral blood of healthy volunteer served as negative controls with unmethylated promotor, while a positive control was clearly methylated.

### Effect of cilengitide and temozolomide on glioma cells

**Figure 9:**
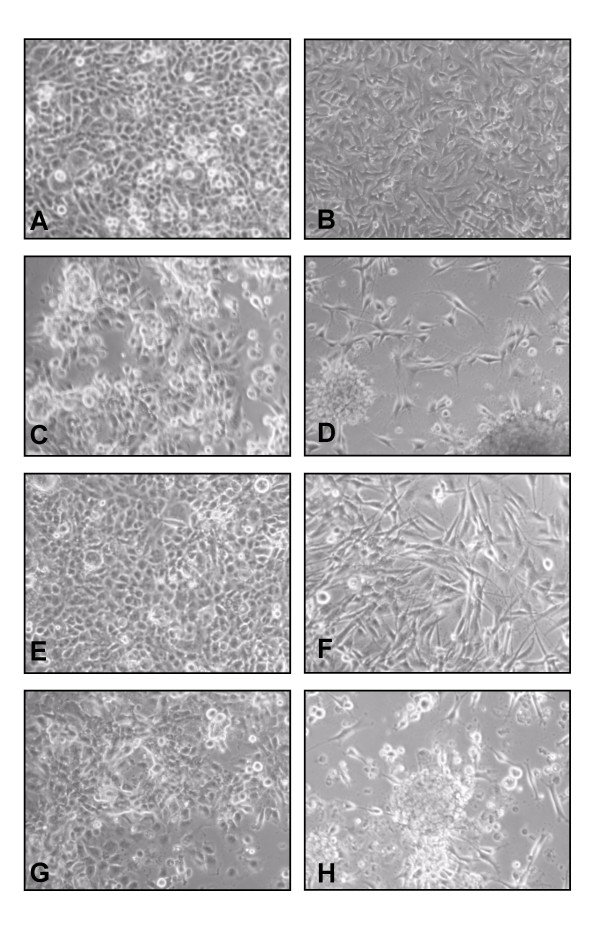
**Effects of cilengitide in combination with temozolomide on cell adhesion and morphology of glioma cells**. Cell lines G28 (left panel) and G44 (right panel) were treated for 24 hours with full medium containing DMSO (A, B), cilengitide at a concentration of 5 μg/ml (C, D), temozolomide (5 μg/ml) (E, F) or cilengitide combined with temozolomide, both at a concentration of 5 μg/ml (G, H) in full medium. Phase contrast micrographs were taken 72 hours after treatment. Results from a representative experiment (of at least 3) are shown.

We next studied the effect of temozolomide (TMZ) in combination with cilengitide on glioma cellls with methylated MGMT promotor. G28 and G44 cells were incubated with cilengitide (5 μg/ml) and temozolomide (5 μg/ml) alone or in combination for 72 hours and changes were studied after 24, 48 and 72 hours. In contrast to cells treated with cilengitide, where many cells detached already after 24 hours (fig. 9C, 9D), G28 (left) and G44 (right) cells treated with TMZ alone did not show morphological changes or cellular detachment when compared to controls (fig. 9E, 9F). The combination of cilengitide and TMZ led to an increased detachment of glioma cells and cell cluster formation being more pronounced after 48 hours (fig 9G, 9H).

### Effect of cilengitide and temozolomide on proliferation and apoptosis of glioma cells

**Figure 10:**
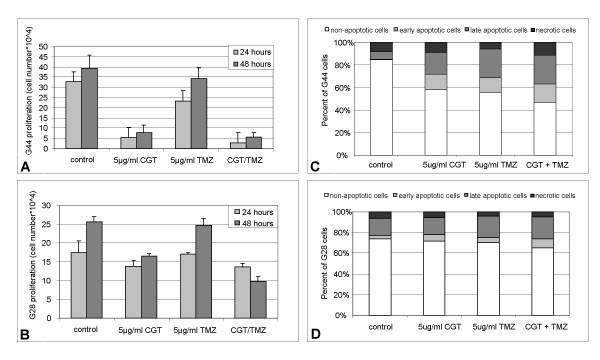
**Cilengitide and temozolomide exert synergistic anti-proliferative and pro-apoptotic effects in glioma cells**. Proliferation. Cell lines G44 (A) and G28 (B) were treated with medium containing 4% FCS and DMSO, cilengitide (5 μg/ml) or/and temozolomide (5 μg/ml). Cells were trypsinized and counted after 24 and 48 hours incubation at 37°C. Apoptosis. Floating and attached G44 (C) and G28 (D) cells were collected 48 hours after treatment with cilengitide and or temozolomide at the indicated concentrations. Apoptosis was assessed by flow cytometry after annexin V and propidium iodide staining. Results from 1 of 4 experiments with similar results are shown.

Glioma cell lines G28 and G44 were treated with 5 μg/ml cilengitide and 5 μg/ml temozolomide alone or in combination and cell counts were determined after 24 and 48 hours. As expected, an inhibitory effect on cell proliferation was observed on glioma cells treated with cilengitide already after 24 hours, whereas treatment with temozolomide alone showed only slight inhibition of proliferation in G44 and G28 glioma cells. When cilengitide was combined with temozolomide proliferation inhibition was slighty pronounced in both cell lines. Quantification of proliferation inhibition by either cilengitide or TMZ alone compared to the combination of both compounds suggested additive effects of cilengitide and TMZ in G44 cells (fig. 10A). The combination caused additional inhibiton in G28 cells. However the data are to limited to propose a synergism of the combination in these cells (fig. 10B). Differences observed between cell lines might be due to higher integrin expression in G44 compared to G28 cells (data not shown).

Annexin V/propidium iodide staining demonstrated similar apoptosis induction after incubation with either cilengitide or temozolomide in G44 cells. The combination of both compounds further increased the amount of apoptotic cells (fig. 10C). Apoptosis induction in G28 cells was less pronounced, but showed similar trends (fig. 10D).

Taken together, these results suggest additive activity of cilengitide combined with TMZ in glioma cells with methylated MGMT promotor.

## Discussion

Experimental data indicated that integrin inhibition using αvβ3 and αvβ5 antagonists may serve as an attractive antiangiogenic therapeutic approach in tumor therapy [34]. Antisense strategies [22], monoclonal antibodies [35] and RGD-related molecules [36,37] have been developed in the previous years and are in various phases of experimental and clinical development [38]. Cilengitide, a polypeptide compound with inhibiting activity on both αvβ3 and αvβ5 integrins has been tested in patients with various advanced solid tumors [39] and profound activity was reported from clinical trials in malignant gliomas [20,21]. For the combination of cilengitide with TMZ the MGMT promotor methylation status seemed to be predictive for therapy response to the combination [21]. In the trial using single agent cilengitide after TMZ failure data regarding MGMT methylation status were not analyzed [20].

Details of the molecular mechanisms of cilengitide in endothelial and glioma cells have not been studied to our knowledge. In our experiments, we observed dose dependent cell rounding and detachment of endothelial cells in tissue culture with cells undergoing apoptosis upon loosing attachment. The morphological changes were accompanied by the loss of intercellular contacts and disorganization of cellular cytoskeleton. Signaling experiments revealed inhibition of integrin-dependent activation of FAK, Src and Akt in two endothelial cell lines, HUVEC and PAE-KDR cells. Cilengitide did not notably interact with KDR-phosphorylation or Erk activation downstream of KDR, although direct interactions between VEGF receptors and integrins have been reported earlier [28,29]. Activity of MAPKinases p38, pJNK and pErk was not altered by cilengitide in HUVEC cells (not shown).

Recently, similar changes have been reported for S 36578-2, a novel RGD mimetic with selective activity on αvβ3 and αvβ5 integrins [40]. This compound induced detachment and was shown to induce apoptosis by direct activation of caspase-8. Since this could be mimicked by culture under non-adhering conditions, the authors stated that anoikis, apoptosis occurring after disruption of cell-matrix interaction, is the underlying mechanism of cell death caused by integrin inhibition.

Earlier data suggested that cilengitide also induces detachment of glioma cells from ECM structures, depending on the matrix used [23]. Further evidence came from other compounds such as contortrostatin, a snake venom disintegrin also based on a RGD-polypeptide [41] and another synthetic RGD-peptide [37] both inducing apoptosis of integrin-expressing glioma cells. Using human glioma cell lines expressing αvβ3 and αvβ5 integrins cilengitide caused a profound detachment and increase of apoptosis in glioma cells similar to what was seen in endothelial cells suggesting that identical mechanisms might occur in both cell types. Indeed, signaling through FAK, Src and Akt was inhibited in the same fashion as observed in endothelial cells.

Tumstatin a collagen IV cleavage product, which has been described as antiangiogenic *in vitro *and *in vivo *acts through inhibition of αvβ3 integrin in endothelial cells [42]. Interestingly, tumstatin was also shown to inhibit growth of glioma cells. Similar to cilengitide its activity is mediated through Akt [43]

Cilengitide induced proliferation inhibition and apoptosis induction in cell lines with methylated MGMT promotor, a predictive factor for responsivness to alkylating agents such as TMZ. TMZ alone was only slightly active in these cells in the concentration used. The combination of both agents did improve response in respect to proliferation and apoptosis compared to cilengitide alone. These results confirm, that cilengitide is active in glioma cells with methylated MGMT promotor as shown in a clinical trial investigating the combination of cilengitide and TMZ [21]. Interestingly, synergistic effects of cilengitide and TMZ have been recently shown for melanoma [44].

## Conclusion

Our data demonstrate molecular and morphological changes induced by cilengitide in integrin-expressing endothelial and glioma cells leading to disruption of cellular contacts and induction of apoptosis/anoikis. Whether the *in vivo *effect of cilengitide is restricted to glioma cells or both endothelial and tumor cells is not clear yet and should be further investigated in order to understand the activity of cilengitide in malignant glioma and help to further improve treatment of this entity.

## Competing interests

GS has a research grant from Merck Serono. JN, IC are employed at Merck Serono.

## Authors' contributions

LOF participated in design of experiments, carried out Western blot analyses, FACS analyses and drafted the manuscript. JH performed proliferation assays and immunostaining. WF participated in design of experiments and drafted manuscript. CB participated in design of experiments and drafted manuscript. JN participated in MGMT methylation assay design. IC participated in design of experiments and drafted manuscript. GS participated in design of experiments, performed microscopical analyses and drafted manuscript.
